# A machine‐learning approach for predicting impaired consciousness in absence epilepsy

**DOI:** 10.1002/acn3.51647

**Published:** 2022-09-16

**Authors:** Max Springer, Aya Khalaf, Peter Vincent, Jun Hwan Ryu, Yasmina Abukhadra, Sandor Beniczky, Tracy Glauser, Heinz Krestel, Hal Blumenfeld

**Affiliations:** ^1^ Department of Neurology Yale University School of Medicine New Haven Connecticut USA; ^2^ Biomedical Engineering and Systems, Faculty of Engineering Cairo University Giza Egypt; ^3^ Department of Clinical Neuorophysiology Danish Epilepsy Center Dianalund Denmark; ^4^ Aarhus University Hospital Aarhus Denmark; ^5^ Division of Neurology Cincinnati Children's Hospital Medical Center Cincinnati Ohio USA; ^6^ Department of Pediatrics University of Cincinnati College of Medicine Cincinnati Ohio USA; ^7^ Epilepsy Center University Hospital Frankfurt Frankfurt Germany; ^8^ Center for Personalized Translational Epilepsy Research (CePTER) Goethe University Frankfurt Germany; ^9^ Department of Neuroscience Yale University School of Medicine New Haven Connecticut USA; ^10^ Department of Neurosurgery Yale University School of Medicine New Haven Connecticut USA

## Abstract

Behavior during 3–4 Hz spike‐wave discharges (SWDs) in absence epilepsy can vary from obvious behavioral arrest to no detectible deficits. Knowing if behavior is impaired is crucial for clinical care but may be difficult to determine without specialized behavioral testing, often inaccessible in practice. We aimed to develop a pure electroencephalography (EEG)‐based machine‐learning method to predict SWD‐related behavioral impairment. Our classification goals were 100% predictive value, with no behaviorally impaired SWDs misclassified as spared; and maximal sensitivity. First, using labeled data with known behavior (130 SWDs in 34 patients), we extracted EEG time, frequency domain, and common spatial pattern features and applied support vector machines and linear discriminant analysis to classify SWDs as spared or impaired. We evaluated 32 classification models, optimized with 10‐fold cross‐validation. We then generalized these models to unlabeled data (220 SWDs in 41 patients), where behavior during individual SWDs was not known, but observers reported the presence of clinical seizures. For labeled data, the best classifier achieved 100% spared predictive value and 93% sensitivity. The best classifier on the unlabeled data achieved 100% spared predictive value, but with a lower sensitivity of 35%, corresponding to a conservative classification of 8 patients out of 23 as free of clinical seizures. Our findings demonstrate the feasibility of machine learning to predict impaired behavior during SWDs based on EEG features. With additional validation and optimization in a larger data sample, applications may include EEG‐based prediction of driving safety, treatment adjustment, and insight into mechanisms of impaired consciousness in absence seizures.

## Introduction

When examining electroencephalography (EEG) recordings, the most apparent marker of generalized epilepsy syndromes is large‐scale synchronous activity, which in the case of absence epilepsy comes as spike‐wave discharges (SWDs) among other activity patterns.^1^ These discharges are typically characterized by spike–wave rhythms with frequencies greater than 2.5 Hz and are often induced clinically via hyperventilation, photic stimulation, or sleep deprivation. It is widely believed that the SWD complexes arise out of thalamocortical oscillations which yield the episodes of impaired consciousness seen in absence epilepsy.^2^, ^3^

A crucial dilemma arises for many people with generalized epilepsy who reach a stage where they are considered clinically seizure‐free but continue to have epileptiform discharges including SWDs on EEG.^4^ EEG discharges with no obvious clinical deficits pose a great challenge to clinicians evaluating a patient's ability to drive or complete other activities affecting the quality of life.^5^ Although they may be imperceptible, epileptiform discharges can result in sudden and transient subtle lapses in cognitive function, which may greatly affect the patient and potentially bring about a public safety concern.^4^, ^6^

Recent work evaluating the behavioral impairment of patients afflicted with absence epilepsy during these discharges has shed some light on various characteristics of EEG and fMRI recording that might be indicative of a lapse in consciousness.^7^, ^8^ To build on the findings of these recent studies, we present an EEG‐based machine‐learning approach that can be used in the future as a clinical tool to effectively predict behavioral impairment.

## Materials and Methods

### Data acquisition

#### Overall data set description

EEG and behavioral data collected in four different studies that focused on absence epilepsy were employed in this work. In particular, two of these studies were conducted at Yale,^7^, ^8^ and the other two were performed by our collaborating institutions: the Danish Epilepsy Center^9^ and the Comprehensive Epilepsy Center at the Cincinnati Children's Hospital Medical Center.^10^ We will henceforth refer to these data sets as follows: cohort A (Yale, Guo et al.^7^), cohort B (Yale, Cohen et al.^8^), cohort C (Danish Epilepsy Center, Beniczky et al.^9^), and cohort D (Cincinnati Children's Hospital Medical Center, Glauser et al.^10^). Table 1 shows the clinical and demographic information of patients within each data set. Of these data sets, cohorts A, B, and C have behavioral labeling for each SWD, while the cohort D data set has labeling for patients indicating if they are free of clinical seizures or not, but not for each individual SWD and is subsequently used to validate our model.

**Table 1:** Patient clinical and demographic information.

Labeled data						
Study	Number of Patients	Females	Age, years (Median ± SEM)	Number of SWD	Spared SWD	Impaired SWD
Cohort A^7^	15	7	10.0 ± 1.0	54	28	26
Cohort B^8^	4	4	20.0 ± 14.2	55	52	3
Cohort C^9^	15	10	14.0 ± 1.9	21	1	20

Unlabeled data						
Study	Number of patients	Females	Age, years (Median ± SEM)	Number of SWD	Patients w/o clinical SWD	Patients w/clinical SWD
Cohort D^10^	41	27	7.6 ± 0.3	220	23	18

SEM, standard error of the mean; SWD, spike‐wave discharge.

#### Yale CAE Study 1 (Cohort A)^7^

Patients were recruited subject to the following criteria: age 6–19 years, diagnosis of either childhood or juvenile absence epilepsy according to International League Against Epilepsy classification, and the presence of 3–4 Hz bilateral SWDs with normal background on EEG. Exclusions were for subjects with additional seizure types (myoclonic, tonic–clonic, partial), structural brain abnormalities, or other such neurological disorders.

EEG was recorded using a high‐density 256‐lead cap at a sampling rate of 500 Hz while patients underwent two behavioral measures of attention during absence episodes: continuous performance (CPT) and repetitive tapping task (RTT). For both behavioral testing paradigms, subjects watched letters on a screen, occurring at a rate of 1 Hz. For the CPT assessment, the task was to press a button whenever an “X” appeared on the screen out of a random sequence of letters. In RTT, subjects were merely asked to press the button every time a letter appeared. With either task, more than one target letter often occurred during any individual SWD. Therefore, with either task, an SWD was defined as “spared” if the correct response rate across all stimuli during the SWD was greater than 75% and “impaired” if it was less than 25%.^7^

#### Yale CAE Study 2 (Cohort B)^8^

Inclusion criteria for this study were as follows: (1) age ≥ 15 years, (2) diagnosed with generalized epilepsy, and (3) at least one epileptiform discharge in routine EEG recording taken within a year of recruitment. Furthermore, patients were excluded for any of the following: any clinical seizures in the month leading up to participation (because the study was aimed at asymptomatic patients) or being diagnosed with any other neurological defect which might inhibit driving ability.

The task paradigm for the study involved a realistic driving simulator in conjunction with real‐time monitoring of the patient's EEG recording which was performed using a 128‐electrode cap with a sampling rate of 1000 Hz.

The behavioral test was conducted while the patient drove around a track in the realistic driving simulator. When the onset of an SWD was detected, the experimenters triggered a red oval (akin to a stop sign) to appear on the screen, prompting the driver to slow down and pull over and thus signal their conscious perception of that stimuli. A “spared” response thus corresponded to an appropriate reaction to the stimuli and an “impaired” response was a lack of reaction, as assessed by gas pedal position, brake pedal force, and the car's velocity.^8^

#### Danish Epilepsy Center Study (Cohort C)^9^

The study inclusion criteria required only that the participant demonstrate a history of absence seizures and 3–4 Hz SWD during scalp EEG recording. The study utilized cup electrodes positioned according to either the 10–20 system or the revised IFCN array^11^ with the data collected at sampling rates of either 500 or 256 Hz.

Behavioral testing involved a line of questioning and assessment of the responsiveness of the patient when it was noted on a live recording of EEG that an SWD had begun.^9^ Testing included any of the following during the ictal period: test responsiveness by saying the subject's name or tapping them, test speech comprehension by asking the patient to raise their arms, verbal function by asking them to repeat a few words, responsiveness by asking orientation questions such as “what is your name?” or “where are you?” testing for anomia by presenting them objects from a box, or asking the subject to count or read/write. Once the SWD was complete, patients were asked if he/she remembered which objects, words, or tests were presented.

For the present study, video recordings of behavior during SWDs were viewed by two reviewers (S.B. and H.B.) and ratings were decided by consensus. SWDs in which the subject was entirely responsive to testing were deemed “spared” and those SWDs in which subjects were entirely unresponsive to the above testing were thus “impaired.” The instance where the subject was responsive to some parts of testing and not others were classified as edge cases (e.g., patients who responded after an SWD to stimuli presented during the SWD) and not used in further analysis.

#### Childhood absence epilepsy Multi‐center Study (Cohort D)^10^

The study inclusion criteria required the following (1) childhood absence epilepsy diagnoses as described by the International League Against Epilepsy, (2) bilateral synchronous, symmetric spike–wave complexes (2.7–5 Hz) on EEG with a normal background, (3) at least one EEG‐recorded SWD lasting >3 s in a 1‐h session. The data were collected at 31 different sites, all using a sampling rate of 256 Hz with a standard 10–20 electrode system.

Inclusion in the present study required the presence of SWDs on EEG recordings performed at the 16/20‐week assessment stage. A patient was classified as spared if no clinical seizures were observed by family or caregivers or induced by hyperventilation. Impaired patients had clinical seizures or seizures induced by hyperventilation reported by family or caregivers.

### Data analyses and machine‐learning classification

#### SWD marking and behavioral analysis

EEGs were reviewed for SWD identification and marking by consensus of two reviewers (M.S. and H.B.) in Cz reference, with SWD onset and end times marked within 0.1 s, blinded to behavioral testing. EEG recordings were then exported and analyzed in MATLAB 2019b (MathWorks).

Individual SWDs were labeled according to the behavioral studies described above, with a binary rating of 0 = normal response (spared), or 1 = lack of response (impaired). As noted previously, the marked SWDs from the cohort D data set were not labeled individually, rather the patients were labeled as either without (spared) or with (impaired) clinical seizures.

#### Pipeline for EEG analysis and machine learning

**Figure 1:**
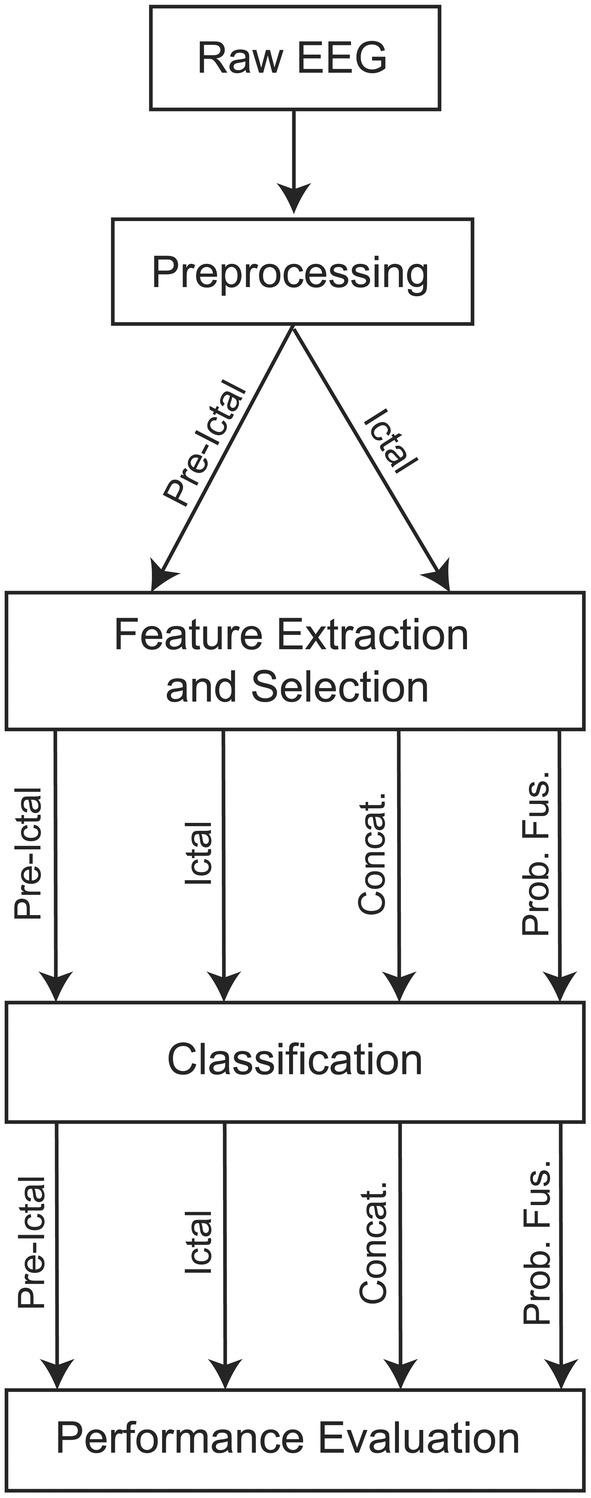
Electroencephalography (EEG) processing pipeline. Workflow diagram of the proposed machine‐learning approach. Preprocessed EEG data corresponding to each seizure was separated into preictal and ictal periods. Different sets of features were extracted independently from preictal and ictal periods, and feature selection was used to identify the significant features. Performance was evaluated based on the preictal window only, the ictal window only, and both preictal and ictal windows (concatenated and probabilistically fused).

A detailed description of the analysis pipeline including preprocessing, feature extraction, feature selection, classification, and performance evaluation is provided in the Data S1 (see Supplementary Materials and Methods in Supporting Information online). These steps are summarized in Figure 1.

#### Overall description of machine‐learning approach

Our objective was to develop a machine‐learning model to predict whether an SWD will yield a lapse in consciousness, defined as impaired responsiveness to external stimuli. Such a predictor could potentially be used for driving safety, so we required that the predictor should never classify SWDs (or patients) as behaviorally spared if in fact they were impaired. Equivalently, we sought a predictor with a zero false discovery rate (or perfect positive predictive value) for spared classification. To be conservative about driving safety, this criterion was considered more important than the sensitivity of the detector. In other words, it was essential to only classify SWD or patients as spared if they were truly spared, even if this was at the expense of not successfully detecting a substantial number of other truly spared SWD or patients.

**Figure 2:**
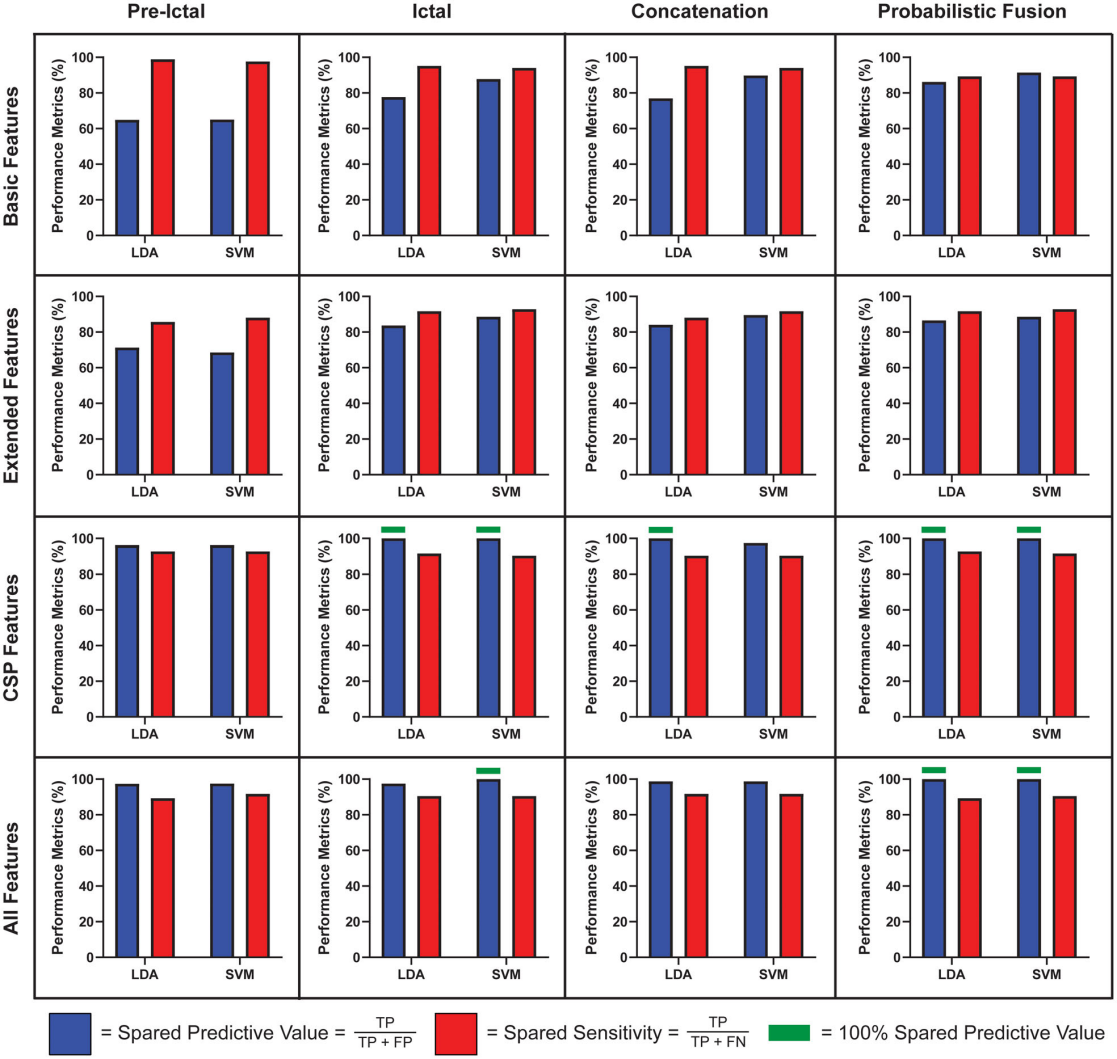
Ten‐fold cross‐validation results with the labeled data sets. Performance was evaluated using 10‐fold cross‐validation on the labeled impaired and spared data (*n* = 130 SWDs) from cohorts A, B, and C. Displayed is the spared predictive value and spared sensitivity from the best‐performing LDA and SVM classifiers for the combinations of feature sets and time windows. Each row corresponds to a feature set and columns represent an EEG time window. Horizontal green bars are indicative of results that achieved the desired performance of 100% spared predictive value. For additional performance metrics see Tables S1–S3. CSP, common spatial pattern; FP, false positive; FN, false negative; LDA, linear discriminant analysis; SVM, support vector machine; TP, true positive.

To achieve this goal, we tested several classifiers with different strategies (Fig. 1). This included classification based on four different types of feature sets (basic features, extended features, common spatial pattern [CSP] features, and all features; see Feature Extraction section in Data S1), and four different combinations of preictal and ictal data (preictal, ictal, concatenation, and probabilistic fusion), fed into either support vector machines (SVM) or linear discriminant analysis (LDA). This resulted in a total of 4 × 4 × 2 = 32 classification models (see Fig. 2).

To determine if preictal or ictal periods or both are needed to achieve the best possible classification performance, we evaluated the performance of the model using the preictal features only, ictal features only, and both preictal and ictal features. Preictal and ictal features were combined using two different approaches. In the first approach, feature vectors of preictal and ictal windows were concatenated to form one feature vector while in the second approach preictal and ictal feature vectors were projected into scalar scores using either LDA or SVM and those scores were combined using weighted Bayesian fusion. This probabilistic fusion approach assumes that the distributions of the preictal and ictal scores are independent but may not have equal importance in determining the severity of the seizure, and therefore, finds the optimal weighting of preictal and ictal feature score distributions to attain correct behavioral classification (see Weighted Probabilistic Bayesian Fusion section in Data S1 for full details).

The classification was developed and tested in two stages. First, the behaviorally labeled data sets from cohorts A, B, and C were used to find the optimal preictal and ictal window sizes and other classifier parameters that maximize the classification performance for individual SWDs. This first classification was done using 10‐fold cross‐validation, by dividing the labeled data repeatedly into training data sets (90% of data) and testing data sets (10% of data). Second, we validated the model performance on the unlabeled data set of patients from cohort D. This was done by training the model on the behaviorally labeled SWDs (cohorts A, B, and C) and then applying the model to the unlabeled SWDs in cohort D.

#### Data and code availability

The data sets generated for this study are available from the corresponding author on reasonable request. The codes generated for the analyses in the study are also available from the corresponding author on reasonable request.

## Results

We first evaluated the performance of the feature sets from the three labeled data sets (cohorts A, B, and C) in which behavioral impairment was known for each SWD, to find the optimal parameters (preictal and ictal window sizes) that maximize the classification performance. Using the optimal set of parameters, we next evaluated the performance of the proposed machine‐learning approach using 10‐fold cross‐validation on the three labeled data sets. Finally, we demonstrated how the proposed approach generalizes on the unlabeled data set from cohort D that was not included in the parameter optimization phase.

### Optimization of preictal and ictal window size

The main objective of the proposed machine‐learning approach is to conservatively label an SWD as spared (potentially safe for driving) only if there truly was no behavioral impairment while at the same time detecting as many spared SWD as possible. Therefore, we sought preictal and ictal window sizes yielding the maximum spared predictive value (equivalent to minimal false discovery rate), while also attempting to maximize sensitivity. Preictal window sizes ranging from 100 to 5000 msec and ictal window sizes ranging from 100 to 1000 msec were tested for the extended feature sets. Window size optimization was not performed on basic features separately because basic features are a subset of the extended features. Preictal windows of 100–1000 msec and ictal windows of 100–500 msec were tested for the CSP features (see Methods). We found that the optimal window size for CSP features was 1000 msec preictal and 500 msec ictal, which yielded corresponding spared predictive value maxima of 100.00% for both intervals. Additionally, we found that the optimal length for the extended feature set was 100 msec preictal and 500 msec ictal, yielding spared predictive values of 80.99% and 83.05%, respectively. However, to be consistent with the optimal findings for the CSP features, we increased the preictal window for the extended features as well to 1000 msec, giving a spared predictive value of 73.90%. Thus, for all subsequent analyses, the basic, extended, and CSP features (and shared features which included all above) were ultimately extracted from the same periods of the EEG recordings for each trial.

### Feature selection

For both basic and extended features, average feature values were calculated (Table 2). To select features for optimal classification, we compared the feature values for behaviorally spared versus impaired SWDs in the labeled data using a Wilcoxon rank‐sum test. A p value criterion of 0.001 yielded the best spared predictive value and sensitivity and was, therefore, used to select features for classification.

**Table 2:** EEG features of behaviorally spared versus impaired SWDs.

Feature	Preictal			Ictal		
	Spared	Impaired	Rank‐sum	Spared	Impaired	Rank‐sum
	(Mean ± SEM)	(Mean ± SEM)	*p*‐Value	(Mean ± SEM)	(Mean ± SEM)	*p*‐Value
Spike power	56.9 ± 4.8	47.6 ± 6.0	0.458	183 ± 18	378 ± 77	0.004
Wave power	27.5 ± 2.6	90.8 ± 15.7	**<0.001**	661 ± 79	2624 ± 403	**<0.001**
SWD duration	–	–	–	947 ± 46	4336 ± 467	**<0.001**
Hjorth activity	200 ± 15	321 ± 42	0.041	924 ± 97	4307 ± 622	**<0.001**
Hjorth mobility	0.14 ± 0.01	0.08 ± 0.00	**<0.001**	0.09 ± 0.00	0.07 ± 0.00	**<0.001**
Hjorth complexity	3.31 ± 0.14	4.08 ± 0.15	**<0.001**	3.75 ± 0.11	3.80 ± 0.16	0.279
Mean Vrms	12.8 ± 0.56	19.1 ± 1.69	0.051	31.6 ± 1.83	57.7 ± 4.83	**<0.001**
Delta power	66.1 ± 6.3	254 ± 44	**<0.001**	859 ± 102	2911 ± 432	**<0.001**
Theta power	39.7 ± 3.8	81.6 ± 15	0.040	435 ± 46	1720 ± 239	**<0.001**
Alpha power	37.5 ± 2.7	42.5 ± 5.8	0.713	144 ± 14	336 ± 60	**<0.001**
Beta power	18.1 ± 1.8	10.8 ± 1.6	0.525	74.3 ± 7.9	128 ± 28	0.009
Low gamma power	4.63 ± 0.49	3.55 ± 0.51	0.388	16.4 ± 1.7	18.2 ± 3.4	0.034
High gamma power	12.5 ± 1.4	4.29 ± 0.66	0.014	16.2 ± 1.7	18.3 ± 3.1	0.522
Voltage mean	−0.64 ± 0.43	−0.92 ± 0.56	0.608	0.37 ± 1.21	3.42 ± 2.57	0.119
Voltage variance	200 ± 15	321 ± 42	0.041	924 ± 97	4307 ± 622	**<0.001**
Voltage skewness	−0.11 ± 0.04	0.03 ± 0.06	0.094	−0.02 ± 0.04	−0.09 ± 0.05	0.351
Voltage kurtosis	3.53 ± 0.1	3.00 ± 0.08	0.005	2.73 ± 0.06	2.47 ± 0.07	0.006
Power mean	0.06 ± 0.01	0.06 ± 0.01	0.561	0.84 ± 0.08	0.79 ± 0.11	0.166
Power variance	0.05 ± 0.01	0.06 ± 0.02	0.172	17.6 ± 2.9	4.84 ± 1.17	0.584
Power skewness	2.52 ± 0.08	2.14 ± 0.10	0.014	1.95 ± 0.06	1.42 ± 0.05	**<0.001**
Power kurtosis	12.1 ± 0.60	9.08 ± 0.66	0.004	7.91 ± 0.35	5.23 ± 0.25	**<0.001**
Multiscale permutation entropy	2.69 ± 0.02	2.56 ± 0.04	0.002	2.39 ± 0.03	2.14 ± 0.04	**<0.001**

Features are calculated for the preictal and ictal windows independently. Results are mean ± SEM for all spared and impaired SWD in the labeled data sets from cohorts A, B, and C. Features with *p* < 0.001 are shown in boldface.

EEG, electroencephalography; SEM, standard error of the mean; SWD, spike‐wave discharge.

As for CSP features, the optimal number of spatial filters that maximize the classification performance was found to be five filters, resulting in 10 CSP features in total (five preictal and five ictal) to be used for classification.

### Performance evaluation of labeled data sets

Using the optimal preictal and ictal window sizes and other parameters as determined above, we tested the model in a 10‐fold cross‐validation scheme using the three labeled data sets, cohorts A, B, and C consisting of 130 SWD in 34 patients (Fig. 2). We used the two key outcome measures, spared predictive value and spared sensitivity, to determine performance for each implementation of the model. We tested both the LDA and SVM classifiers, using four different combinations of preictal and ictal data (preictal only, ictal only, concatenation, probabilistic fusion) and four different types of feature sets (basic, extended, CSP, and all features) to yield a total of 32 classification models (Fig. 2).

The optimal metric of 100.00% spared predictive value was achieved only for classifiers using CSP or all features (bottom two rows of Fig. 2). Considering spared sensitivity, the best overall performance was achieved by classifiers using weighted probabilistic fusion, with feature sets of CSP or all features (right column, bottom two rows). Thus, with probabilistic fusion, a spared predictive value of 100% was achieved together with excellent spared sensitivities for CSP (93% sensitivity for LDA and 91% for SVM) and for all features (89% for LDA and 90% for SVM) (Fig. 2). The optimal preictal window weightings for each of these classifiers were *α* = 0.73 (CSP‐LDA), 0.48 (CSP‐SVM), 0.70 (All‐LDA), and 0.28 (All‐SVM).

The other classifiers which achieved 100% spared predictive value were the following: only the ictal window, CSP features yielded spared sensitivities of 92% for LDA and 90% for SVM; only the ictal window, all features yielded a spared sensitivity of 90% with SVM; and concatenation of the preictal and ictal windows and using CSP features yielded a sensitivity of 90% with LDA (Fig. 2). The detailed performance metrics for all classifiers are presented in Tables S2 and S3.

### Common spatial pattern visualization

**Figure 3:**
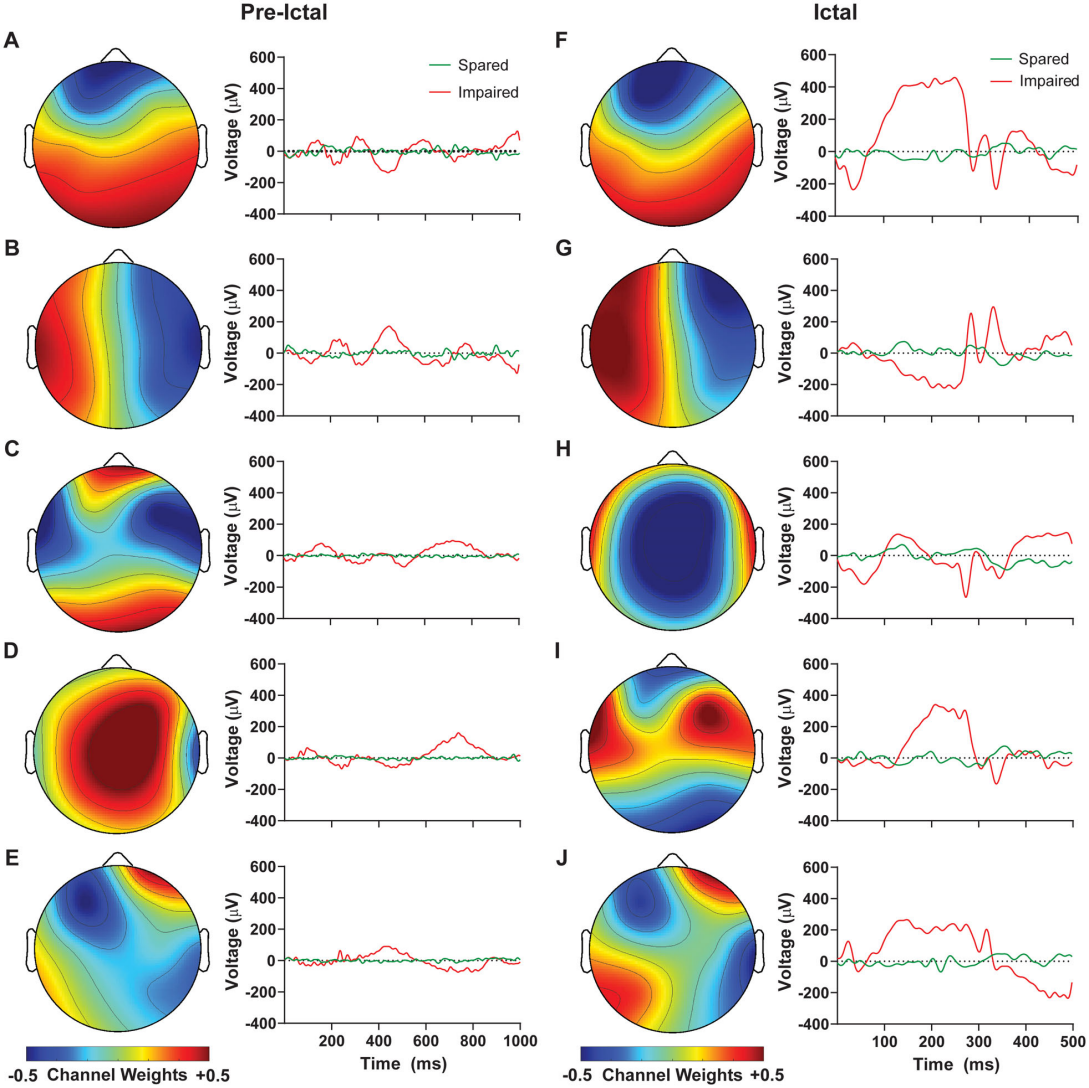
Common spatial pattern (CSP) visualization of features distinguishing spared versus impaired SWD. Top spatial filters were obtained by applying the CSP algorithm on the 1000 msec preictal windows prior to SWD onset (A–E) and the 500 msec ictal windows following SWD onset (F–J). Color topo plots on the left of each panel show spatial filters displayed for the preictal and ictal periods, sorted in descending order in terms of variance, and demonstrate the weightings of each channel in defining each spatial filter. Voltage time courses on the right of each panel show an example of spared SWD and an example of impaired SWD from a single patient projected onto the corresponding spatial filters.

Given the role of the CSP features in the successful classifications obtained either with CSP features or all features (Fig. 2), we were interested in examining the properties of the CSP features that distinguish spared from impaired SWDs. Figure 3 shows the top five spatial filters from the CSP projection matrix used to discern the EEG data for spared and impaired discharges for both the 1000 msec preictal and 500 msec ictal windows. The filters are presented as color topographic plots of channel weights, shown from top to bottom in decreasing order in terms of the percent variance explained between the two classes. These filters demonstrate a clear anterior–posterior, left–right, or outer–inner topographic weighting contributing to the successful classification for both the preictal and ictal time series. For example, the top spatial filter for both preictal and ictal EEG show positive weights for the posterior EEG channels and negative weights for the anterior channels (Fig. 3A and F), implying that the maximum difference in variance between the impaired and spared SWDs can be obtained by applying a transform to the SWDs that positively weight the EEG data samples from posterior electrodes and negatively weights the EEG data samples from anterior electrodes.

**Figure 4:**
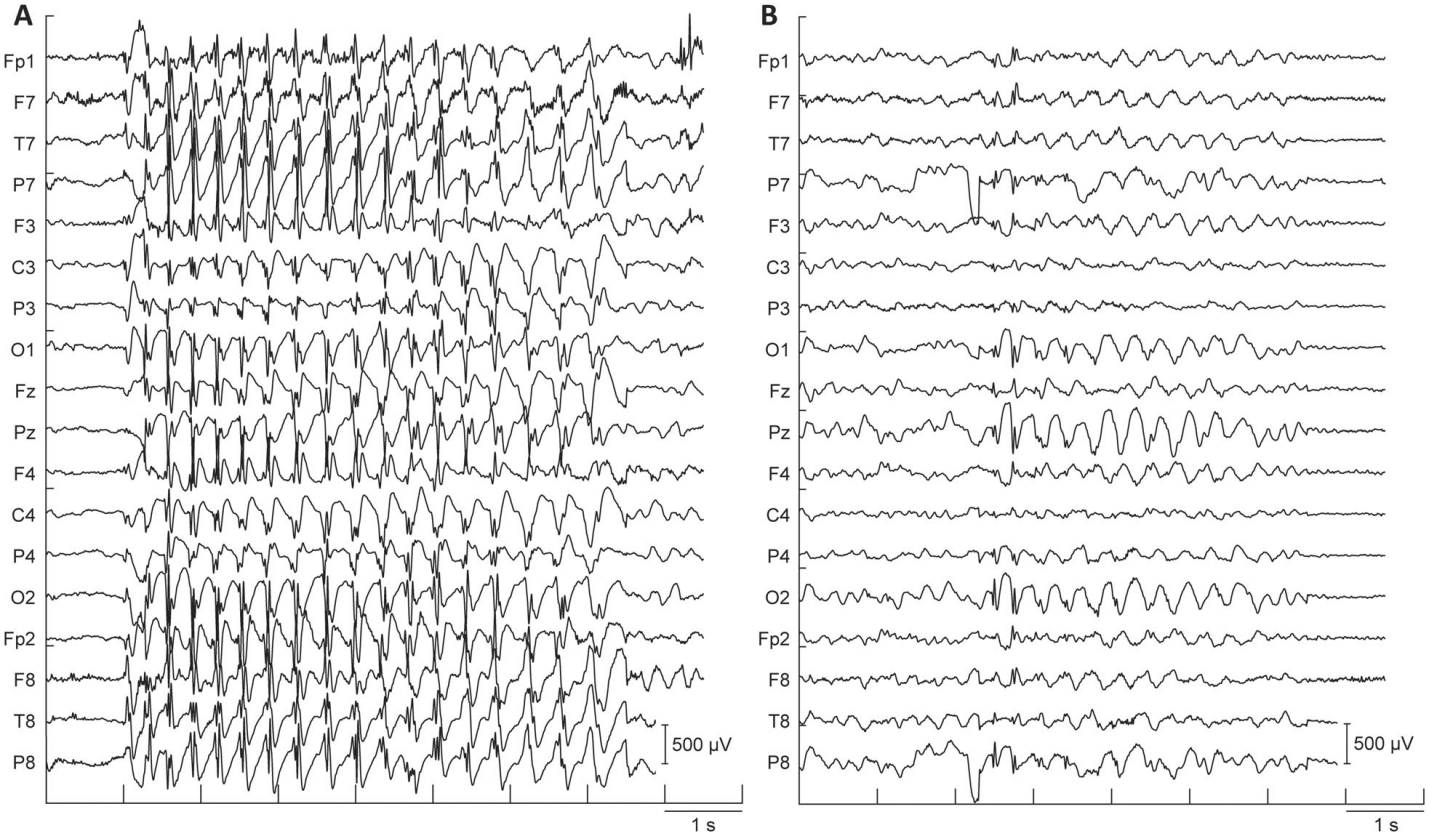
Electroencephalographies of impaired and spared spike‐wave discharges (SWD). Examples of SWD with spared and impaired responses to behavioral testing. Data are displayed on a reduced 16‐channel subset of the 10–20 system used for computational analysis via common spatial pattern methods. Recordings were re‐referenced to a common average reference. The scale bar is 500 μV. (A) An example of an SWD, which was behaviorally classified as impaired and (B) spared. (A) and (B) are the same data as displayed in the projected voltage time course SWD examples in the right panels of Figure 3 and drawn from the same patient.

Additionally, the projections of an example spared and an example impaired SWD for the 1000 msec preictal and 500 msec ictal time windows are displayed in Figure 3. The projected SWD signals were obtained by multiplying the zero‐mean original data by the channel weights for each filter. Examining the projected time courses, we see that the spatial filters identified by CSP maximize the impaired class variance while minimizing the spared. In both the preictal and ictal projections, we see close to baseline time courses for the spared class while the impaired class exhibits high amplitude and rhythmic activity. To provide a more intuitive view of these differences, Figure 4 shows the original example SWDs used to obtain the CSP time course projections in Figure 3, displayed using a standard 10–20 EEG montage. Here we can clearly see the SWD morphology, present in both spared and impaired SWD from the same patient, as well as characteristics that allow for discerning the two. These include the duration, amplitude of rhythmic activity, and topographic distribution of the signals, well captured by the classification model.

### Validation with unlabeled data set

**Figure 5:**
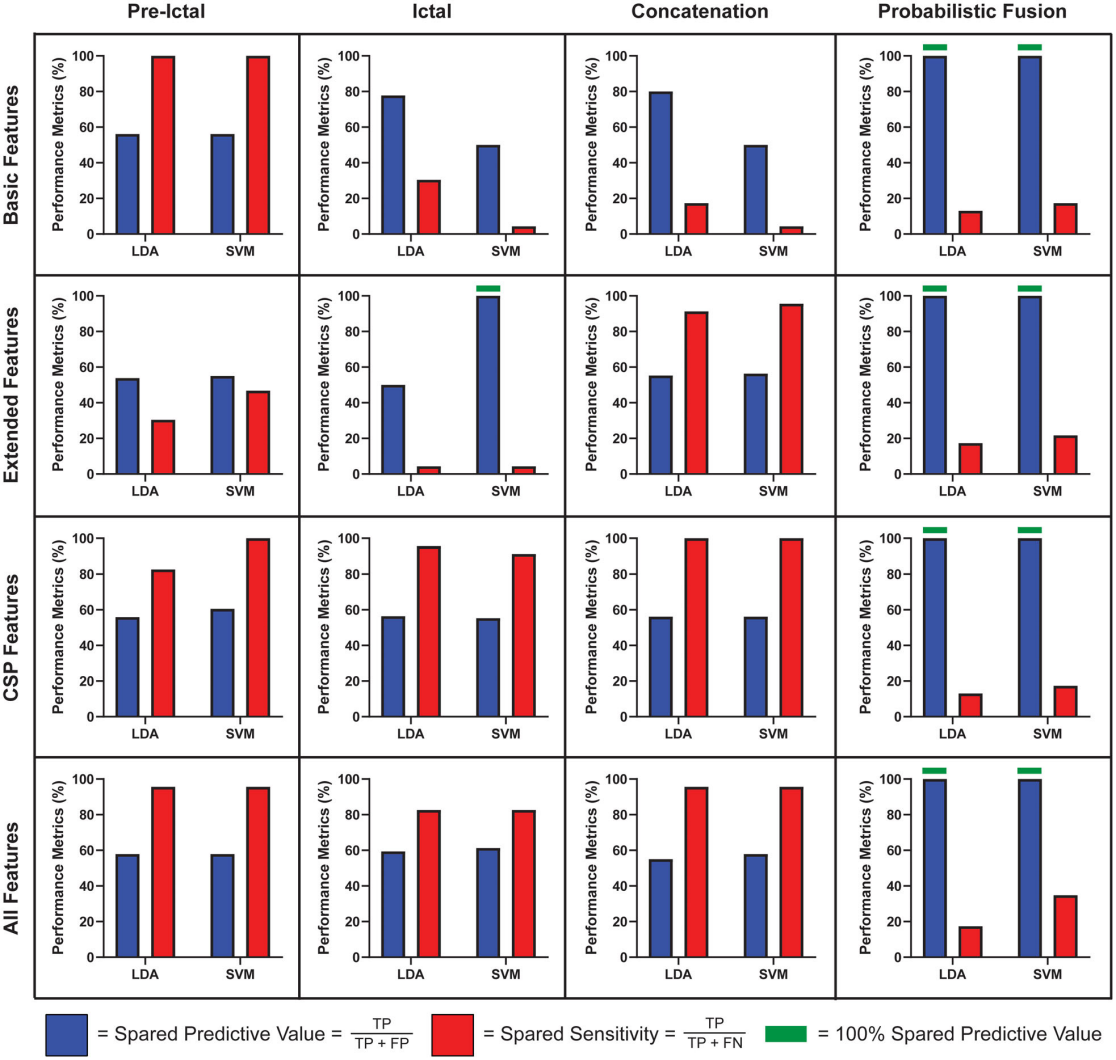
Validation of classifiers on unlabeled data set. Classifiers were optimized with the labeled data (Fig. 2) and applied to the unlabeled data set of cohort D (*n* = 41 patients, 220 SWDs). Displayed are the spared predictive value and spared sensitivity from the LDA and SVM classifiers for all the combinations of feature sets and time windows. Each row corresponds to a feature set and each column represents an EEG time window. Horizontal green bars indicate the combinations that achieved the desired performance of 100% spared predictive value. CSP, common spatial pattern; FP, false positive; FN, false negative; LDA, linear discriminant analysis; SVM, support vector machine; TP, true positive.

We next applied the optimized model from the labeled data sets to an unlabeled data set from cohort D where the behavior for individual SWD was unknown (*n* = 220 SWD in 41 patients). As described in Methods, classification of the unlabeled data was done on the patient level by considering a patient to be impaired if they had any SWD determined by the model to be impaired, and a patient was considered spared only if all their SWD were determined by the model to be spared. These results were compared to observations by family or caregivers who thought the patients were having clinical seizures (18 of 41 patients) or not (23 of 41 patients). Our conservative criterion for classifier success was once again a spared predictive value of 100%, but now at the patient level, meaning that no patient should be classified as spared if in fact they were reported to have clinical seizures. This 100% spared predictive value criterion was achieved mainly by classifiers using weighted probabilistic fusion (Fig. 5, right column). The conservative 100% safety‐oriented criterion, however, came with relatively low sensitivity to detecting spared patients. The most successful classifier with 100% spared predictive value had a spared sensitivity of 35% (8 of 23 spared patients), achieved by SVM with probabilistic fusion and all features (Fig. 5, lower right). In other words, by the conservative criteria used here, when our most successful classifier was applied to the unlabeled SWD in the 23 patients who were considered by family and caregivers to have no clinical seizures, 8 were successfully classified as spared, however, 15 were not classified as “safe” (spared) based on our model. On the other hand, all 18 patients who were observed by patients or caregivers to have clinical seizures were classified by our model as impaired.

## Discussion

For people with absence epilepsy and no obvious behavioral seizures, clinicians currently decide on driving privileges and other factors such as medication adjustment solely based on commonsense EEG characteristics, such as the presence of many SWDs with longer durations.^12^ While previous studies have been successful in identifying EEG and fMRI characteristics that are correlated with behavioral impairment,^7^, ^8^, ^13^ none have attempted to devise a machine learning‐based system that can predict behavioral impairment by learning from labeled seizure examples as originally proposed by Antwi et al.^4^ Here we introduce a promising machine‐learning approach that can be built upon in the future for use as a clinical tool to accurately assess the risk of behavioral impairment in those diagnosed with absence epilepsy but without obvious clinical episodes. Using EEG data, our approach successfully predicted spared behavioral responsiveness with a false discovery rate of zero (positive predictive value of 100%) for four different data sets. Our goal was to minimize the false discovery rate because if such a tool is used in the future to evaluate driving safety, it is imperative to eliminate the possibility of incorrectly classifying an impaired patient as free from clinical seizures.

Considering the 10‐fold cross‐validation analyses performed on the three labeled data sets with behavioral testing, the most basic feature set (spike power, wave power, and SWD duration) achieved a maximum spared predictive value of 98% with a spared sensitivity of 65%. Inclusion of additional features in the “extended” feature set led to a maximum spared predictive value of 93% but with an increase in sensitivity up to 89%. The use of CSP‐based features alone or in conjunction with the extended feature set as “all features,” yielded the best performance, particularly when using a weighted probabilistic fusion of preictal and ictal data, providing a positive predictive value of 100% and sensitivity of up to 93%. Thus, the machine‐learning approach introduced in this work was highly successful in correctly classifying SWD for which behavioral responsiveness was known based on ictal behavioral testing.

In previous studies, the CSP algorithm has been used predominantly for the purposes of brain–computer interfacing and motor control studies.^14^, ^15^, ^16^, ^17^ In this work, we have extended the applications of CSP to include EEG analysis of absence epilepsy. Examining the CSP analysis outcomes, the time course projections retain some rhythmic activity and spike‐wave‐like dynamics, especially for the impaired class, consistent with prior work showing larger amplitude for SWD with impaired behavior.^7^, ^8^, ^13^ In addition, the top spatial filters retain spatial information that aligns well with the literature on absence epilepsy. In particular, for both the preictal and ictal time periods, CSP identifies a strong localizing component to discern the two classes, which aligns well with literature arguing against the uniform generalized spatial distribution of SWDs.^18^, ^19^ It is also of interest that the identified spatial filters for both the preictal and ictal windows have significant overlap in terms of channel weightings which could be indicative of similar spatial activity before, during, and after a discharge and could thus be predictive of the onset itself.^20^

In validating our model on the unlabeled data from cohort D, we see once again that the inclusion of CSP features in the all features‐based classification using probabilistic fusion yielded to the best classifier. This classifier was hesitant to label a patient as entirely spared when experiencing discharges but was absolutely confident when electing to make such a decision (zero false discovery rate/100% spared predictive value). Such a classification approach is optimal as a clinical tool as it would ease restrictions only on patients who are predicted to retain consciousness awareness and responsiveness during a discharge with high confidence.

Although we have achieved an optimal value of 100% spared predictive value corresponding to a false discovery rate of zero in validating our model on unlabeled data, the sensitivity of identifying patients with spared behavior in the unlabeled data was quite low (35%). Of note, the patients felt to be spared of clinical absence seizures based on observations by family and caregivers did have SWD, but these were not formally assessed for more subtle behavioral impairment. Such incomplete characterization of the behavioral effects of SWDs might explain the low sensitivity of the model in identifying patients with spared behavior. Moreover, it is possible that some of the patients considered to be free of absence seizures by family and caregivers, but classified as impaired by our model, might in fact have had behavioral impairments that would make driving hazardous during SWD. To investigate this possibility further, additional investigation will be needed in a larger cohort, and with behavioral testing during SWD in all participants. The decreased sensitivity of our classifier in detecting spared patients might also be due to the relatively small sample size of the labeled data sets used for training the proposed model. In particular, the selected features for classification as well as the spared and impaired patterns learned by the classifiers for this data set may not generalize very well to the larger population due to overfitting the small training data set and thus can result in a degradation in some of the metrics determining model performance when applied to a separate patient group. In addition, the fact that the data sets have clinically notable differences because the patients in these data sets have different epilepsy syndromes could be a factor in the degraded model performance due to the existence of various SWD patterns with a small number of training examples per each pattern. In case a large data set is used to train the model, this heterogeneity is expected to yield a classifier with better generalizability as it will be trained on various patterns that are represented by a sufficient number of training examples. Generally, a large sample size that is more representative of SWD global characteristics is expected to allow improved spared sensitivity and overall model performance.

Additionally, a large sample size would allow the use of more robust classification approaches that are expected to have better generalizability such as deep‐learning approaches that require large training data sets to perform efficiently. Previously, convolutional neural networks have been used for similar EEG analyses including predicting seizure onset.^20^, ^21^, ^22^ Instead of using hand‐crafted features, deep‐learning approaches automatically learn features from the data to distinguish the classes of interest. This will allow the discovery of relevant features that were not identified before by human observers. A drawback of these approaches, however, is the loss of explainability in modeling. In other words, deep‐learning features that are extracted automatically may be difficult to interpret in relation to the biological processes driving seizure activity. Common sense features, such as SWD duration, magnitude, and frequency of occurrence, have been examined previously in relation to behavioral impairment,^7^, ^8^, ^12^, ^13^ and more rigorous identification of these and possibly additional predictive features would be highly clinically useful.

Another future direction that has the potential to improve classification performance across patients is to use transfer learning for identifying the optimal training data to be used with each subject. This would involve devising a metric for measuring similarity between the data of each subject in the training set and the data in the test set and train on those patients who have the highest similarity with the patient being tested. The methodology would overcome obstacles pertaining to data variability across subjects and sessions within a subject.^23^

Although fMRI data are in a sense more difficult and time‐consuming to obtain than a scalp EEG, and thus less efficient as a clinical tool, they could provide greater insight into the underlying mechanisms of impaired consciousness.^7^, ^24^, ^25^, ^26^, ^27^ In conjunction with EEG data, a more precise machine‐learning model might be developed, although cost and general availability of fMRI may be prohibitive for many people with epilepsy.

Future work might also include the generalization of our approach to more broad diagnoses outside of childhood and juvenile absence epilepsy. The present study focuses only on the SWD and its characteristics, however, many other forms of epilepsy exist with a plethora of EEG markers such as poly‐spike wave activity, rhythmic slowing or higher frequency rhythmic activity. Epilepsy is characterized by large‐scale synchronous behavior in the brain and as a result, more advanced machine‐learning strategies should be implemented on a comprehensive data set to pinpoint more generalized characteristics of lapses in consciousness. With additional future work, hopefully, such approaches will increase understanding of epilepsy pathophysiology and lead to better practical guidance that will improve the quality of life for people with epilepsy.

## Conflict of Interest

The authors declare that they have no competing financial interests.

## Supporting information


**Data S1.** Supplementary Materials and Methods.
**Table S1.** Performance metric definitions.
**Table S2.** Performance of support vector machine Classifier for all Feature Sets and Temporal Windows.
**Table S3.** Performance of linear discriminant analysis Classifier for all Feature Sets and Temporal Windows.
