# Supplementary material for: A machine‐learning approach for predicting impaired consciousness in absence epilepsy
Source: Ann Clin Transl Neurol. 2022 Sep 16;9(10):1538–50. doi: 10.1002/acn3.51647 (PMC9539371; doi:10.1002/acn3.51647)
Supplement: Supplementary file 1 — Data S1. Supplementary Materials and Methods. Table S1. Performance metric definitions. Table S2. Performance of support vector machine Classifier for all Feature Sets and Temporal Windows. Table S3. Performance of linear discriminant analysis Classifier for all Feature Sets and Temporal Windows. [file ACN3-9-1538-s001.docx]

***Supplementary Materials for:***

**A Machine Learning Approach for Predicting Impaired Consciousness in Absence Epilepsy**

^1,*^Max Springer, ^1,2,*^Aya Khalaf, ^1^Peter Vincent, ^1^Jun Hwan Ryu, ^1^Yasmina Abukhadra, ^3^Sandor Beniczky, ^4,5^Tracy Glauser, ^1,6,7^Heinz Krestel, ^1,8,9^Hal Blumenfeld,

^1^Yale University School of Medicine, Department of Neurology, New Haven, CT, USA

^2^Biomedical Engineering and Systems, Faculty of Engineering, Cairo University, Giza Egypt

^3^Danish Epilepsy Center, Department of Clinical Neuorophysiology, Dianalund, and Aarhus University Hospital, Aarhus, Denmark

^4^Cincinnati Children’s Hospital Medical Center, Division of Neurology, Cincinnati, OH, USA ^5^University of Cincinnati College of Medicine, Department of Pediatrics, Cincinnati, OH, USA

^6^University Hospital Frankfurt, Epilepsy Center, Frankfurt, Germany

^7^Goethe University, Center for Personalized Translational Epilepsy Research (CePTER), Frankfurt, Germany

^8^Yale University School of Medicine, Department of Neuroscience, New Haven, CT, USA

^9^Yale University School of Medicine, Department of Neurosurgery, New Haven, CT, USA

*These authors contributed equally to this work

**Supplementary Materials and Methods**

In the sections below, we provide a detailed description of the analysis pipeline including EEG preprocessing, feature extraction, feature selection, classification, and performance evaluation as depicted in Figure 1.

*EEG Preprocessing*

EEG signals were preprocessed using EEGLAB (Swartz Center for Computational Neuroscience, University of California, San Diego) toolbox. Signals were resampled to 500 Hz (EEGLAB) to reduce computational complexity and maintain consistency across datasets. To remove baseline drift, a finite impulse response (FIR) high-pass filter with a corner frequency of 0.1 Hz was used. Line noise was removed by CleanLine (EEGLAB) which adaptively estimates and removes sinusoidal noise from EEG channels using multi-taper regression and a Thompson F-statistic to identify significant sinusoids from 50/60 Hz noise. Individual EEG recordings were then visually inspected to remove leads with obvious movement artifacts. To further reduce artifacts, we excluded edge electrodes positioned off the scalp. In addition, we excluded any one second data epoch from the recording where the absolute voltage exceeded a defined threshold: 150% of the absolute voltage across leads of the first two seconds in each SWD, averaging voltage first within channel then across channels. Finally, a peri-ictal period of up to 20s before SWD onset and up to 20s after offset was then defined for each discharge and extracted, defining the remaining data outside of the peri-ictal window as baseline. For SWDs that were within 20s of one another, we evenly divided the inter-ictal window into post and pre-ictal periods for the two events. A common average reference was employed to re-reference the peri-ictal and ictal period by subtracting the average baseline potential of all channels from the peri-ictal data at each channel.

Recent studies have demonstrated some EEG characteristics in the time prior to the start of an SWD, such as beta and theta rhythm power[^1^](#_ENREF_1), as being correlated with the duration and intensity of an episode. Therefore, for each SWD, we segmented both the period preceding the SWD onset (pre-ictal window) and the period during which the SWD occurs (ictal window). In particular, the pre-ictal window consisted of time samples corresponding to up to 20s prior to SWD onset while the ictal window comprised the time points from SWD onset to offset.

*Overall Description of Machine Learning Approach*

Our objective was to develop a machine learning model to predict whether an SWD will yield a lapse in consciousness, defined as impaired responsiveness to external stimuli. Such a predictor could potentially be used for driving safety, so we required that the predictor should never classify SWDs (or patients) as behaviorally spared if in fact they were impaired. Equivalently, we sought a predictor with a minimum false discovery rate (or maximum positive predictive value) for spared classification. To be conservative about driving safety, this criterion was considered more important than sensitivity of the detector. In other words, it was essential to only classify SWD or patients as spared if they were truly spared, even if this was at the expense of not successfully detecting a substantial number of other truly spared SWD or patients.

To achieve this goal, we tested several classifiers with different strategies (Figure 1). This included classification based on four different types of feature sets (see Feature Extraction section below), four different combinations of preictal and ictal data, fed into either support vector machines (SVM) or linear discriminant analysis (LDA). This resulted in a total of 4 × 4 × 2 = 32 classification models (see Figure 2).

To determine if pre-ictal or ictal periods or both are needed to achieve the best possible classification performance, we evaluated the performance of the model using the pre-ictal features only, ictal features only, and both pre-ictal and ictal features. Pre-ictal and ictal features were combined using two different approaches. In the first approach, feature vectors of pre-ictal and ictal windows were concatenated to form one feature vector while in the second approach pre-ictal and ictal feature vectors were projected into scalar scores using either LDA or SVM and those scores were combined using weighted Bayesian fusion (see Weighted Probabilistic Bayesian Fusion section below).

The classification was developed and tested in two stages. First, the behaviorally labeled datasets from Guo et al.[^2^](#_ENREF_2) (cohort A), Cohen et al.[^3^](#_ENREF_3) (cohort B), and Beniczky et al.[^4^](#_ENREF_4) (cohort C), were used to find the optimal pre-ictal and ictal window sizes and other classifier parameters that maximize the classification performance for individual SWDs. This first classification was done using 10-fold cross validation, by dividing the labeled data repeatedly into training data sets (90% of data) and testing data sets (10% of data). Second, we validated the model performance on the unlabeled dataset of patients from Glauser et al.[^5^](#_ENREF_5) (cohort D). This was done by training the model on the behaviorally labeled SWDs (cohorts A, B, and C) and then applying the model to the unlabeled SWDs in cohort D data.

*Feature Extraction*

We extracted four sets of features from both the pre-ictal and ictal windows including basic, extended, and common spatial pattern (CSP) based feature sets as well as a feature set that includes all the previously mentioned features. The basic and extended features assume that channels are independent in the temporal and frequency domain, whereas the CSP features do not restrict the data to such an assumption.

*Feature Sets 1 and 2: Basic and Extended Features*

Initially, for each SWD, a singular value decomposition (SVD) was applied to the ictal time series to cluster channels that have similar spike-wave form and polarity and reject channels that do not correlate with the any other channel and thus are not useful in characterizing the discharge. We elected to use SVD because it produces components that are equivalent to the output of principal component analysis (PCA) but avoids having to compute a covariance matrix, thus reducing the overall complexity [^6-9^](#_ENREF_6). By extracting exemplary traces via SVD, we reduce the total number of pairwise correlations that need to be computed. Parameters for the clustering included an initial thresholding parameter of 0.01 times the largest singular value, a correlation threshold of 0.8 to assign components to the same cluster, and a correlation threshold of 0.5 to reject channels from further analysis. For the ictal period, features were extracted for each channel within the largest non-rejected cluster, and then each feature was averaged across channels. The selected channels for feature extraction were then used for the pre-ictal EEG time series as well for consistency and each feature was again averaged across channels. The following features, unless otherwise noted, were calculated for both the pre-ictal and ictal time series.

We defined the “basic” feature set as the spike power (10 - 125 Hz), wave power (2.5 – 4Hz), and SWD duration in milliseconds, motivated by prior studies relating these features to impaired behavior in SWD[^2^](#_ENREF_2). The band power in these frequency ranges was calculated by estimating the power spectral density (PSD) and calculating the area under PSD curve in the frequency region of interest.

The extended feature set encompasses the basic feature set as well as features describing statistical and frequency domain characteristics of the SWDs as detailed below.

PSD-based band power was calculated in the following biologically relevant frequency ranges: 1 – 4 Hz (delta), 2.5 – 4 Hz (wave), 4 – 8 Hz (theta), 8 – 15 Hz (alpha), 15 – 30 Hz (beta), 30 – 45 Hz (low gamma), 45 – 100 Hz (high gamma) and 10 – 125 Hz (spike). The four moments – mean, variance, skewness, and kurtosis – are widely used in classifying time courses[^10^](#_ENREF_10) and were calculated for the PSD of each channel. These moments were also calculated in a relativistic manner where the pre-ictal moment was divided by its corresponding ictal moment.

The Hjorth parameters[^11^](#_ENREF_11) — activity, mobility, and complexity — were calculated per signal and convey general frequency analysis information through general time domain characteristics. The activity is the variance of the signal, the mobility assesses the dominant frequency in the signal, and the complexity describes the change in frequency of the signal by showing how the shape of the signal compares to a pure sine wave — a complexity equal to 1 being representative of the sine wave. Mean, skewness, and kurtosis were also calculated as above but in the time domain. Variance in the time domain was excluded as it is equivalent to the Hjorth activity.

In accordance with previous studies correlating simple EEG features to impairment, the root mean square voltage (V_RMS_) was calculated per channel[^3^](#_ENREF_3)^,^ [^12^](#_ENREF_12). Lastly, the rhythmicity of SWDs, which has been noted as leading to more severe impairment [^12^](#_ENREF_12) was quantified using the multiscale permutation entropy[^13^](#_ENREF_13)^,^ [^14^](#_ENREF_14). This nonlinear statistic identifies self-similarity within a signal by determining the probability and relative frequency of recurrent patterns within the time-series data.

*Feature Sets 3 and 4: Common Spatial Pattern (CSP) Features and All Features*

The CSP algorithm[^15^](#_ENREF_15) was originally devised to discriminate patients with abnormal EEG from healthy subjects in a population of scalp EEG data, and has since been used in epilepsy studies on scalp EEG data for predicting seizure onsets with low computational complexity[^16^](#_ENREF_16)^,^ [^17^](#_ENREF_17). The objective of the CSP algorithm is to find the optimal spatial filters that linearly transform observations of a two-class problem (spared versus impaired) to a new multidimensional space in which the two classes are more separable in terms of variance yielding a significant improvement for the two-class learning problem. When applied to spared and impaired SWD observations, the spatial filters will project the EEG into a space which maximizes the variance for one class (either spared or impaired SWDs) while minimizing the variance for the other class simultaneously.

In this work, we applied CSP filtering on pre-ictal and ictal time courses separately. When finding the optimal spatial filters, we selected only a common subset of 16 channels from the standard 10-20 electrode signal for all recordings to conserve the spatial topography across patients from different collection sites. The CSP feature set is henceforth defined as the log of the variance of the projections[^18^](#_ENREF_18) of each SWD’s pre-ictal and ictal time course onto the corresponding spatial filters.

We additionally define a fourth feature set which comprises all features described above, including CSP, basic and extended and we refer to it as the “all features” set.

*Feature Selection and z-scoring*

For the basic and extended feature sets, a non-parametric Wilcoxon rank-sum test was conducted to select features with statistically significant differences between the spared and impaired SWD in cohorts A, B, and C data sets. The Wilcoxon rank-sum test was favored over the Student t-test because the latter is a parametric test and assumes the features follow a Gaussian distribution, which is not a reasonable assumption considering our small sample size[^19^](#_ENREF_19).

Feature selection was done separately for the two main analyses (classification of individual SWDs; and classification of patients) with similar results. For the classification of individual SWDs in the labeled data sets, features were selected for each iteration of the 10-fold cross validation by comparing spared versus impaired SWD in the labeled training data set (90% of the 130 SWDs). For the classification of patients, features were selected from the entire labeled data set (all 130 SWDs). The performance was evaluated at three different p-values, 0.05, 0.01 and 0.001 and the p-value criterion of 0.001 was chosen for all subsequent analyses because it gave the best performance based on positive predictive values and sensitivity. Features selected by these criteria are summarized in the Results (see also Table 2).

For the selected basic and extended features, each feature in the individual SWD episodes was then z-scored using the average value and standard deviation of that feature calculated across all SWD from the labeled training data set observations.

As for CSP, selecting features that are most relevant to the classification problem was conducted by choosing the number of spatial filters in the pre-ictal and ictal periods that maximize the classification performance, again based on positive predictive value and sensitivity.[^20^](#_ENREF_20)

*Classification*

The performance of the proposed model was evaluated using LDA[^21^](#_ENREF_21) and SVM[^22^](#_ENREF_22) classifiers. LDA is a linear classifier that assumes the observations belonging to each class follow a multivariate Gaussian distribution. SVM does not restrict the data to such an assumption, and it seeks to find the optimal hyperplane such that the distance between that hyperplane and the nearest observation from the training data is maximized. These classifiers were used directly to classify pre-ictal features only, ictal features only, and concatenated pre-ictal and ictal features. Moreover, for the probabilistic fusion of the pre-ictal and ictal features, LDA and SVM were used to project pre-ictal and ictal features into scalar scores as explained in detail in the next section below.

*Weighted Probabilistic Bayesian Fusion* One of the methods for combining the pre-ictal and ictal feature sets is the weighted probabilistic Bayesian fusion as originally implemented in the combination of EEG and functional transcranial Doppler ultrasound (fTCD) data[^18^](#_ENREF_18). This Bayesian approach relies on the assumption that the pre-ictal and ictal distributions are independent but may not have equal weighting in determining the severity of the seizure and thus making the correct behavioral classification.

Probabilistic fusion was applied to the four feature sets described above independently using LDA and SVM classifiers. For each feature set, we projected the pre-ictal and ictal feature vectors separately into two scalar scores, derived either from an LDA or SVM classifier. For the LDA classifier, scores correspond to the probability that an observation belongs to a class (spared or impaired). For SVM, the score is the distance from the decision boundary, where positive and negative values correspond to belonging to each class. For each class, a probability distribution was estimated from the training data for the pre-ictal and ictal scores separately. For example, the probability distribution for a particular feature set in the preictal period for either LDA or SVM in the impaired class consists of all the scalar scores for that feature set across SWD in the impaired class in the training data. These pre-ictal and ictal probability distributions were then weighted by weights α and 1- α, respectively where α ranged between 0 and 1, and for each class (spared, impaired), the two weighted distributions were multiplied following the assumption that the pre-ictal and ictal distributions are independent but may not have equal weighting in determining the severity of the seizures. Consequently, each class was described by one probability distribution that when fed a test observation of unknown classification, yields a probability that the input belongs to that class. The test observations were assigned to the class (spared or impaired) yielding the maximum probability. Optimal weight α was obtained by sweeping the parameter in the range $[0,1]$ to maximize performance, evaluated by positive predictive value and sensitivity for spared SWD as in the original work by Khalaf et al.[^18^](#_ENREF_18)^,^ [^23^](#_ENREF_23) Selection of weights was done separately for each feature set and class, for LDA and SVM.

*Performance Assessment*

To evaluate the performance of the proposed model under the different combinations of feature sets, classification methods, time windows and other parameters, the predicted labels of the test feature vectors were compared to the true labels assigned in the behavioral studies for each combination to produce the following metrics.

Considering that the “positive” class refers to spared SWDs and “negative” refers to impaired SWDs, then a true positive (TP) is thus a SWD that was labeled as spared by our classifier and was also labeled as spared in behavioral testing, a true negative (TN) is the same for an impaired SWD and the false positive (FP) and false negative (FN) are SWDs which were labeled incorrectly by our classifier. Using these definitions, we calculate standard performance metrics for accuracy, class sensitivity and class predictive value (see Table 3). Of the above, we emphasize the spared predictive value (SPV, 1 – the false discovery rate) and sensitivity as they are most clinically relevant for the purposes of our study. As was already discussed (Overall Description of Machine Learning Approach), by maximizing these metrics, we can ensure with high confidence that patients who are predicted to be free of behavioral impairment are safe to carry out tasks such as driving.

*Optimization of Pre-ictal and Ictal Window Size*

To determine the optimal window size to be used for extracting each feature set, we evaluated the performance metrics described earlier (see Performance Assessment section), as a function of the EEG window size being used, for both the pre-ictal and ictal windows to identify the length of time which yields a maximal SPV.

Because the extended features already encompass the basic features, we assessed performance over time separately for only the extended and CSP feature sets using 10-fold cross validation on the available labeled datasets (cohorts A, B, and C). Moreover, since the “all features set” encompasses the extended and CSP features, we did not search for an optimal window size for that set. Due to its low computational complexity compared to SVM, LDA was used to evaluate the performance over time for the different combinations of feature sets (extended and CSP) and time windows (pre-ictal and ictal).

Performance was evaluated at time points starting at 100ms up to 5000ms for the pre-ictal window and up to 1000ms for the ictal window with 100ms increments. At each time point, if the pre-ictal or ictal periods of a given SWD were shorter than the time length that this time point corresponds to, the entire window of that SWD was used for this time point and all the following time points. As for CSP, the features are limited in that all trials must be of equal length, and as a result we only extract these features up to the length of our shortest pre-ictal period (1000ms) and ictal period (500ms).

*Performance Evaluation on Labeled Datasets*

All the SWD for which we had behavioral testing data and thus impaired/spared labeling were used to assess the performance of the proposed approach. The datasets used here are the cohort A, B, and C data sets. Training and test sets were created using 10-fold cross validation (n = 130 SWD in 34 patients). For each of the 10-fold iterations, 10% of the SWD were randomly selected as the test set and the remaining 90% were used as the training set. The 10% of SWD used for the test set were distinct for each iteration, such that all 130 SWDs were ultimately tested. For each iteration, we used the training data to develop an optimal LDA or SVM model used to predict the behavioral labeling of each corresponding set of test data. After combining all the predicted labels across all tested data sets, we then compared these to the actual labeling from behavioral testing and assessed the accuracy on the SWD level. This means that each predicted SWD labeling was treated as one observation which was either a TP, TN, FP, or FN. These results were then used to calculate positive predictive value and sensitivity for spared SWDs across the entire labeled data set for each classifier.

*Clinical Validation on Unlabeled Dataset*

To validate our model on an unlabeled dataset, we instead evaluated the performance on a patient-by-patient basis (n = 41 patients) rather than on an SWD level. We trained the model using the data from cohorts A, B, and C and included all 130 SWDs as the training set. We then used the classifiers obtained this way to predict the labels of the SWDs for each patient from cohort D study. Since each patient in this study was described as either having clinical seizures (what we would describe as impaired behavioral responsiveness) or not (spared), we used the following strict criterion to assess the accuracy of our classification. Patients were classified as spared if and only if all the recorded SWDs were labeled as spared by our classifier, and impaired if at least one of their SWDs was classified as impaired. As a result, a TP occurred if our model classified all of a patient’s SWDs as spared and the physicians or families noted no clinical seizures, a TN if any SWDs were classified by our model as impaired and the physicians or families noted clinical seizures were present; and similar criteria were used to determine FP and FN.

*Data and Code Availability*

The datasets generated for this study are available from the corresponding author on reasonable

request. The codes generated for the analyses in the study are also available from the corresponding author on reasonable request.

**References**

1. Sorokin JM, Paz JT, Huguenard JR. Absence seizure susceptibility correlates with pre-ictal beta oscillations. J Physiol Paris 2016;110:372-381.

2. Guo JN, Kim R, Chen Y, et al. Impaired consciousness in patients with absence seizures investigated by functional MRI, EEG, and behavioural measures: a cross-sectional study. The Lancet Neurology 2016;15:1336-1345.

3. Cohen E, Antwi P, Banz BC, et al. Realistic driving simulation during generalized epileptiform discharges to identify electroencephalographic features related to motor vehicle safety: Feasibility and pilot study. Epilepsia 2020;61:19-28.

4. Beniczky S, Neufeld M, Diehl B, et al. Testing patients during seizures: A European consensus procedure developed by a joint taskforce of the ILAE - Commission on European Affairs and the European Epilepsy Monitoring Unit Association. Epilepsia 2016;57:1363-1368.

5. Glauser TA, Cnaan A, Shinnar S, et al. Ethosuximide, valproic acid, and lamotrigine in childhood absence epilepsy. N Engl J Med 2010;362:790-799.

6. Tanwar S, Ramani T, Tyagi S. Dimensionality Reduction Using PCA and SVD in Big Data: A Comparative Case Study. 2018;220:116-125.

7. Wall ME, Rechtsteiner A, Rocha LM. Singular Value Decomposition and Principal Component Analysis. In: Berrar DP, Dubitzky W, Granzow M, eds. A Practical Approach to Microarray Data Analysis. Boston, MA: Springer US, 2003: 91-109.

8. Simek K, Fujarewicz K, Świerniak A, et al. Using SVD and SVM methods for selection, classification, clustering and modeling of DNA microarray data. Engineering Applications of Artificial Intelligence 2004;17:417-427.

9. Horn D, Axel I. Novel clustering algorithm for microarray expression data in a truncated SVD space. Bioinformatics 2003;19:1110-1115.

10. Hasan MK, Ahamed MA, Ahmad M, Rashid MA. Prediction of Epileptic Seizure by Analysing Time Series EEG Signal Using k-NN Classifier. Appl Bionics Biomech 2017;2017:6848014.

11. Hjorth B. EEG analysis based on time domain properties. Electroencephalography and Clinical Neurophysiology 1970;29:306-310.

12. Mirsky AF, Van Buren JM. On the nature of the “absence” in centrencephalic epilepsy: A study of some behavioral, electroencephalographic and autonomic factors. Electroencephalography and Clinical Neurophysiology 1965;18:334-348.

13. Ouyang G, Li J, Liu X, Li X. Dynamic characteristics of absence EEG recordings with multiscale permutation entropy analysis. Epilepsy Res 2013;104:246-252.

14. Bandt C, Pompe B. Permutation entropy: a natural complexity measure for time series. Phys Rev Lett 2002;88:174102.

15. Koles ZJ, Lazar MS, Zhou SZ. Spatial patterns underlying population differences in the background EEG. Brain Topogr 1990;2:275-284.

16. Zheng G, Yu L, Feng Y, et al. Seizure prediction model based on method of common spatial patterns and support vector machine. 2012: 29-34.

17. Zhang Y, Guo Y, Yang P, Chen W, Lo B. Epilepsy Seizure Prediction on EEG Using Common Spatial Pattern and Convolutional Neural Network. IEEE J Biomed Health Inform 2020;24:465-474.

18. Khalaf A, Sejdic E, Akcakaya M. Common spatial pattern and wavelet decomposition for motor imagery EEG- fTCD brain-computer interface. J Neurosci Methods 2019;320:98-106.

19. Blair RC, Higgins JJ. A Comparison of the Power of Wilcoxon's Rank-Sum Statistic to that of Student'st Statistic Under Various Nonnormal Distributions. Journal of Educational Statistics 2016;5:309-335.

20. Blankertz B, Tomioka R, Lemm S, Kawanabe M, Muller K-r. Optimizing Spatial filters for Robust EEG Single-Trial Analysis. IEEE Signal Processing Magazine 2008;25:41-56.

21. Fisher RA. The Use of Multiple Measurements in Taxonomic Problems. Annals of Eugenics 1936;7:179-188.

22. Cortes C, Vapnik V. Support-vector networks. Machine Learning 1995;20:273-297.

23. Khalaf A, Akcakaya M. A probabilistic approach for calibration time reduction in hybrid EEG-fTCD brain-computer interfaces. Biomed Eng Online 2020;19:23.

**Supplementary Tables**

**Supplementary Table e1. Performance Metrics Definitions**

|  | $Accuracy (ACC)=\frac{TP+TN}{P+N}$ |  |
| --- | --- | --- |
|  | $Spared Sensitivity (SSN)=\frac{TP}{TP+FN}$ |  |
|  | $Impaired Sensitivity (ISN)=\frac{TN}{TN+FP}$ |  |
|  | $Spared Predictive Value (SPV)=\frac{TP}{TP+FP}$ |  |
|  | $Impaired Predictive Value (IPV)=\frac{TN}{TN+FN}$ |  |

As explained in the main text, the “positive” class refers to spared SWDs and “negative” refers to impaired SWDs. Thus, a true positive (TP) is a SWD that was labeled as spared by our classifier and was also labeled as spared in behavioral testing; a true negative (TN) is a SWD that was labeled as impaired by our classifier and was also labeled as impaired SWD impaired in behavioral testing; and the false positive (FP) and false negative (FN) are SWDs which were labeled incorrectly by our classifier.

**Supplementary Table e2.** Performance of SVM Classifier for all Feature Sets and Temporal Windows

|  |  | Pre-Ictal | Ictal | Concatenated | Probabilistic Fusion |
| --- | --- | --- | --- | --- | --- |
| Basic Features | SPV | 65.08 | 87.78 | 89.77 | 91.45 |
|  | SSN | 97.62 | 94.05 | 94.05 | 89.29 |
|  | IPV | 50.00 | 87.50 | 88.10 | 81.25 |
|  | ISN | 04.35 | 76.09 | 80.43 | 84.78 |
|  | ACC | 64.62 | 87.69 | 89.23 | 87.69 |
| Extended Features | SPV | 68.52 | 88.64 | 89.53 | 88.64 |
|  | SSN | 88.10 | 92.86 | 91.67 | 92.86 |
|  | IPV | 54.55 | 85.71 | 84.09 | 85.71 |
|  | ISN | 26.09 | 78.26 | 80.43 | 78.26 |
|  | ACC | 66.15 | 87.69 | 87.69 | 87.69 |
| CSP Features | SPV | 96.30 | 100.00 | 97.44 | 100.00 |
|  | SSN | 92.86 | 90.48 | 90.48 | 91.67 |
|  | IPV | 87.76 | 85.19 | 84.62 | 86.79 |
|  | ISN | 93.48 | 100.00 | 95.65 | 100.00 |
|  | ACC | 93.04 | 93.85 | 92.31 | 94.62 |
| All Features | SPV | 97.47 | 100.00 | 98.72 | 100.00 |
|  | SSN | 91.67 | 90.48 | 91.67 | 90.48 |
|  | IPV | 86.72 | 85.19 | 86.54 | 85.19 |
|  | ISN | 95.65 | 100.00 | 97.83 | 100.00 |
|  | ACC | 93.08 | 93.85 | 93.85 | 93.85 |

SPV = spared predictive value, SSN = spared sensitivity, IPV = impaired predictive value, ISN = impaired sensitivity,

ACC = accuracy (Supplementary Table e1 for full definitions)

**Supplementary Table e3.** Performance of LDA Classifier for all Feature Sets and Temporal Windows

|  |  | Pre-Ictal | Ictal | Concatenated | Probabilistic Fusion |
| --- | --- | --- | --- | --- | --- |
| Basic Features | SPV | 64.84 | 77.67 | 76.92 | 86.21 |
|  | SSN | 98.81 | 95.24 | 95.24 | 89.29 |
|  | IPV | 50.00 | 85.19 | 84.62 | 79.07 |
|  | ISN | 02.17 | 50.00 | 47.83 | 73.91 |
|  | ACC | 64.62 | 79.23 | 78.46 | 83.85 |
| Extended Features | SPV | 58.62 | 81.58 | 76.19 | 82.93 |
|  | SSN | 85.71 | 91.67 | 88.10 | 91.67 |
|  | IPV | 58.62 | 81.58 | 76.19 | 82.93 |
|  | ISN | 36.96 | 67.39 | 69.57 | 73.91 |
|  | ACC | 68.46 | 83.08 | 81.54 | 85.38 |
| CSP Features | SPV | 96.30 | 100.00 | 100.00 | 100.00 |
|  | SSN | 92.86 | 91.67 | 90.48 | 92.86 |
|  | IPV | 87.76 | 86.79 | 85.19 | 88.46 |
|  | ISN | 93.48 | 100.00 | 100.00 | 100.00 |
|  | ACC | 93.08 | 94.62 | 93.85 | 95.38 |
| All Features | SPV | 97.40 | 97.44 | 98.72 | 100.00 |
|  | SSN | 89.29 | 90.48 | 91.67 | 89.29 |
|  | IPV | 83.02 | 84.62 | 86.54 | 83.64 |
|  | ISN | 95.65 | 95.65 | 97.83 | 100.00 |
|  | ACC | 91.54 | 92.31 | 93.85 | 93.08 |

SPV = spared predictive value, SSN = spared sensitivity, IPV = impaired predictive value, ISN = impaired sensitivity,

ACC = accuracy (see Supplementary Table e1 for full definitions)
